# Extraction, Structural Characterisation, and Immunomodulatory Properties of Edible *Amanita hemibapha* subspecies *javanica* (Corner and Bas) Mucilage Polysaccharide as a Potential of Functional Food

**DOI:** 10.3390/jof7090683

**Published:** 2021-08-24

**Authors:** Utoomporn Surayot, Sutee Wangtueai, SangGuan You, Subramanian Palanisamy, Warawut Krusong, Charles S. Brennan, Francisco J. Barba, Yuthana Phimolsiripol, Phisit Seesuriyachan

**Affiliations:** 1 College of Maritime Studies and Management, Chiang Mai University, Samut Sakhon 74000, Thailand; utoomporn.su@cmu.ac.th (U.S.); sutee.w@cmu.ac.th (S.W.); 2 Department of Marine Food Science and Technology, Gangneung-Wonju National University, Gangwon 210-702, Korea; umyousg@gwnu.ac.kr (S.Y.); spalanisamy33@gwnu.ac.kr (S.P.); 3 East Coast Life Sciences Institute, Gangneung-Wonju National University, Gangwon 210-720, Korea; 4 Division of Fermentation Technology, Faculty of Food Industry, King Mongkut’s Institute of Technology Ladkrabang, Bangkok 10520, Thailand; warawut.kr@kmitl.ac.th; 5 School of Science, STEM College, RMIT University, Melbourne 3000, Australia; charles.brennan@rmit.edu.au; 6 Department of Preventive Medicine and Public Health, Food Science, Toxicology and Forensic Medicine, Faculty of Pharmacy, Universitat de València, Avda. Vicent Andrés Estellés s/n, 46100 Burjassot, Spain; francisco.barba@uv.es; 7 Faculty of Agro-Industry, Chiang Mai University, Chiang Mai 50100, Thailand; yuthana.p@cmu.ac.th; 8 Cluster of Agro Bio-Circular-Green Industry, Chiang Mai University, Chiang Mai 50100, Thailand; 9 Advanced Manufacturing and Management Technology Research Center (AM2Tech), Department of Industrial Engineering, Faculty of Engineering, Chiang Mai University, Chiang Mai 50200, Thailand

**Keywords:** mucilage polysaccharide, mushroom, *Amanita hemibapha* subspecies *javanica* (Corner and Bas), immunomodulatory property, fractionation, functional food

## Abstract

This research aimed to extract mucilage polysaccharides (MP) from *Amanita hemibapha* subspecies *javanica* (Corner and Bas), and further fractionate them using anion-exchange chromatography, yielding two fractions (MPF1 and MPF2). The crude extract, and fractions mainly consisted of carbohydrates (83.5–93.2%) with minor amounts of proteins (5.40–7.20%), and sulphates (1.40–9.30%). Determination of the monosaccharide composition revealed that glucose was the major unit, followed by galactose, mannose, rhamnose, and arabinose. The average molecular weight (MW) of the crude extract and fractions was in the range 104.0–479.4 × 10^3^ g/mol. Interestingly, the crude extract, and fractions did not cause any toxic effect in RAW264.7 cells. However, they stimulated the RAW264.7 cells to release nitric oxide and cytokines through the activation of nuclear factor-kappa B (NF-κB), and mitogen-activated protein kinase (MAPK) pathways via cell surface TLR4. Structural analysis of the most immunestimulating extract fraction, MPF2, revealed that the main backbone consisted of α-D-(1→6)-glucopyranoside. These results suggest that the MPs derived from *A. hemibapha* subspecies *javanica* (Corner and Bas) are potent in enhancing immunity; hence, they can be used as a functional ingredient in food products.

## 1. Introduction

Mushrooms are special groups of fungi, and there may be a particular growth of mushroom species associated with the seasons. Mushrooms are considered a healthy food, being a great source of minerals, dietary fibre, and vitamins [1], which can be applied in pasta [2], meat [3], and bread for enhancing the nutritional properties [4]. There has been recent interest in mushrooms not only to satisfy hunger or provide nutrition, but also as a source of biologically active compounds of medicinal value. Moreover, their uses include complementary medicine or dietary supplements for anticancer, antiviral, immunostimulatory, hepatoprotective, and hypocholesterolaemic agents, which has urged a global market for natural foods consumed as dietary supplements [5].

*Amanita hemibapha* subspecies *javanica* (Corner and Bas) is an edible mushroom, and one of the most popular wild mushrooms in Thailand. This species belongs to the genus Amanita, which grows in the north, and north-east regions of Thailand where it is traditionally gathered, and consumed as a food. The mushrooms have their own special taste, and a distinctive flavour. Usually, the fungus, and its host, such as insects, are mostly consumed in various countries including Japan, China, and Korea because it contains high nutritional benefits such as anti-ageing, immunomodulatory, and anticancer properties as well as encourages improvement of renal function [6,7,8]. The existence of chemical constituents such as polysaccharides, proteins, mannitol, fatty acids, and trace elements may be responsible for many biological functions of the fungus [9]. *A. hemibapha* subspecies *javanica* secretes polysaccharide-rich mucilage when cooked or in contact with water. The mucilage is a thick, slimy polysaccharide, and is used to thicken soups, and stews. It is a common component of plants such as okra (*Abelmoschus esculentus*) [10], aloe (*Aloe vera*) [11], linseed (*Linum usitatissimum*) [12], and rice bran (*Oryza sativa* L.) [13,14]. A previous study showed the great potential of the bioactive components of *A. hemibapha* subspecies *javanica* to scavenge the hydroxyl radical, being, for instance, an excellent source of antioxidant compounds [15].

Nowadays, polysaccharides from fungi have attracted great attention in biochemical, and nutritional studies due to their potential in the development of new functional foods, and nutraceuticals. Many studies have reported that polysaccharides from mushrooms have a variety of types of bioactivities such as immunomodulatory, antioxidant, anticancer, antibacterial, and anti-inflammatory bioactivities, as shown in Table 1. This table shows extraction methods, and the bioactivity of polysaccharides from mushrooms [16,17,18,19,20,21,22,23,24]. The immunostimulatory activity is due to the great potential of polysaccharides such as lentinan, schizophyllan, and β-glucan to modulate the immune system [25]. In the immune system, macrophages play a role in innate immunity, serving as a first line to protect against pathogens, and tumours. Moreover, macrophages also connect the innate, and adaptive immunity. It has been reported that the polysaccharide β-glucan from the edible mushroom *Entoloma lividoalbum* acts as an immune-enhancing agent via macrophage, splenocyte, and thymocyte cells, and also exhibits antioxidant activity [26]. Treatment with VGPI—a polysaccharide isolated from *Volvariella*—showed an immunostimulatory effect on RAW264.7 cells by enhancing the mRNA expression of NO, and cytokines, and also showed upregulation via the mitogen-activated protein kinase (MAPK) signalling pathway [27]. Another in vitro study on the immunomodulatory activity of polysaccharides from *Cantharellus cibarius* Fr. showed that they have an immunomodulatory effect by inducing phagocytosis, and higher levels of NO, and cytokines [16]. The immunomodulatory activity is influenced by the chemical composition, molecular weight, structure, branching, configuration, and conformation of polysaccharides [28,29].

However, there are no studies evaluating the molecular structures, and bioactivity of mucilage polysaccharides from *A. hemibapha* subspecies *javanica* in the available literature. Therefore, in the present work, the mucilage polysaccharides from the *A. hemibapha* subspecies *javanica* fruiting body were extracted using hot water extraction, and fractionated by anion-exchange chromatography. Structural analysis was carried out using gas chromatography–mass spectrometry (GC–MS) to determine the monosaccharide composition, and glycosidic linkage. Meanwhile, high-performance size exclusion chromatography was used for measurement of molecular weight, and both 1D, and 2D nuclear magnetic resonance (NMR) analyses were used for assessing the structure. Furthermore, RAW264.7 cell activation was tested by various in vitro assays to confirm the immunostimulatory properties of the polysaccharide, which can be further applied as a functional food ingredient in food, and beverage products as well as in dietary supplements.

## 2. Materials and Methods

### 2.1. Raw Materials, and Reagents

The fruiting bodies of the wild mushroom *A. hemibapha* subspecies *javanica* were purchased from the local market in Chiang Mai, Thailand, in 2018. They were carefully washed with tap water, and dried using a hot air oven at 60 °C for 3 h. The dried sample was finely milled with a grinder into powder, and stored at −20 °C. RPMI-1640 cell culture medium, fetal bovine serum (FBS), streptomycin, and penicillin were purchased from Gibco Life Technologies (Grand Island, NY, USA). Griess reagent was purchased from Sigma-Aldrich (NSW, Australia). The EZ-Cytox cell viability assay kit (WST-1) was purchased from Daeil Lab Service Co., Ltd., Korea. The anti-complement receptor 3 antibody (anti-CR3), and anti-toll-like receptor 4 antibody (anti-TLR4) were obtained from Abcam (Cambridge, MA, USA). The phospho-NF-κB antibody, phospho-p38 antibody (MAPK), phospho-ERK (MAPK) antibody, and phospho-JNK antibody were purchased from Cell Signaling Technology (Danvers, MA, USA). All other chemicals, and reagents used in this study were of high analytical grade.

### 2.2. Mucilage Polysaccharide Extraction

Five hundred grams of dried mushroom powder was dissolved in 1000 mL of distilled water. The mixture was then heated at 60 °C under constant stirring for 1 h. The concentrated solution was cooled to room temperature, and filtrated through a muslin cloth. Approximately 20 mL of acetone was added to the concentrated solution. The mucilage was filtered through a muslin cloth, and cooled again. Trichloroacetic acid (80%) was added to the supernatant, which was then stored at 4 °C for 30 min, and centrifuged (4000 rpm for 10 min). The supernatant was mixed with three volumes of ethanol (EtOH, 99%), and stored at 4 °C overnight to precipitate the crude mucilage polysaccharides (MPs). The obtained MPs were thoroughly washed with EtOH, and dried at room temperature. The yield of the isolated polysaccharides was calculated in relation to the depigmented powder obtained after the 99% EtOH treatment.

### 2.3. Fractionation of MPs

Briefly, MPs (150 mg) were dissolved in distilled water (10 mL), and the solution was injected into a DEAE–Sepharose fast flow column (17–070–01; GE Healthcare Bio-Science AB, Uppsala, Sweden) equilibrated with distilled water. The polysaccharides were eluted with distilled water to obtain a neutral polysaccharide, and subsequently eluted with a stepwise NaCl gradient (0.5–2.0 M) at a flow rate of 1.5 mL/min; the polyanionic polysaccharides were then collected. The obtained fractions were identified by phenol–sulphuric acid assay, and all fractions were extensively dialysed (3K MWCO) against distilled water for 3 days, and then lyophilised.

Two fractions were obtained, referred to as mucilage polysaccharide fraction 1 (MPF1), and mucilage polysaccharide fraction 2 (MPF2).

### 2.4. Chemical Composition of Polysaccharides

The phenol–sulphuric acid method was employed to determine the sugar content, using D-glucose as a reference [30]. The protein content was evaluated by the Lowry method using a Bio-Rad DC Protein assay kit [30]. The sulphate content was calculated by hydrolysing the polysaccharide with 0.5 M HCl, and then following the BaCl_2_ gelatin method using K_2_SO_4_ as a standard [31]. The uronic acid content was determined by a sulphamate/m-hydroxydiphenyl assay using glucuronic acid as a standard [32]. The average molecular weight (MW), and radius of the gyration (*Rg*) of the polysaccharides were determined using a high-performance size exclusion chromatography column (TSK G5000PW column) coupled to UV, multi-angle laser light scattering, and a refractive index detection system (HPSEC-UV-MALLS-RI) according to a previously described method [33].

### 2.5. Analysis of Monosaccharide Composition, and Absolute Configuration

The monosaccharide composition was determined by GC following reduction, and acetylation. Briefly, samples (3 mg) were hydrolysed with 4 mL of 4 M trifluoracetic acid (TFA) at 100 °C for 6 h. Excess TFA was removed with methanol, and the mixture was reduced through the addition of sodium borodeuteride (NaBD_4_) solution. Acetylation was conducted with acetic anhydride to give alditol acetates, which were determined by GC–MS (6890N/MSD 5973, Agilent Technologies, Santa Clara, CA, USA) using an HP–5MS capillary column. Monosaccharide standards (arabinose, xylose, rhamnose, mannose, glucose, and galactose) were used as references. The absolute configuration of the monosaccharide was analysed by GLC with acetylated (R)-2-methylheptyl glycosides as described previously [34].

### 2.6. Methylation Analysis

Methylation analysis was carried out to determine the types of linkage between residues according to the method of Cao et al. (2014) with slight modifications. The polysaccharide (2–3 mg) was dissolved in 0.5 mL dimethyl sulphoxide (DMSO). Methyl iodide (CH_3_I) was added for methylation. The methylated polysaccharides were hydrolysed with 4 M TFA at 100 °C for 6 h. The hydrolysates were then reduced in distilled water with NaBD_4_, and acetylated with acetic anhydride. The acetylated derivatives were extracted with methylene chloride. The partially methylated alditol acetates were analysed by GC–MS using methods previously published by Cao et al. (2014) [31].

### 2.7. NMR Spectroscopy

The NMR spectra were obtained from the sample (10 mg) after desulphation that was dissolved in D_2_O (0.5 mL). The ^1^H, and ^13^C NMR spectra of the sample were recorded on a JEOL ECA-600 spectrometer (JEOL, Akishima, Japan) at 70 °C at a base frequency of 150 MHz for ^13^C, and 600 MHz for ^1^H. Two-dimensional COSY, and HMQC experiments were processed using the pulse programs.

### 2.8. RAW264.7 Macrophage Proliferation, and Nitric Oxide (NO) Production Assay

In the macrophage cell proliferation assay, RAW264.7 cells were determined according to the WST-1 method. The RAW264.7 cells (100 µL) were plated into a 96-well plate at 1 × 10^6^ cells/well in RPMI-1640 medium containing 10% FBS. The samples (MP, MFP1, and MPF2) were added at different concentrations (50, 100, and 200 µg/mL), and the medium was used as the control. The 96-well plate was incubated at 37 °C with 5% CO_2_ for 24 h. After incubation, 10% WST-1 solution was added to each well, and the plate was further incubated for 1 h. The absorbance was determined using a microplate reader, and measured at 450 nm (EL-800; BioTek Instruments, Winooski, VT, USA).

The NO production assay was conducted by the Griess method [35]. In brief, RAW264.7 cells (1 × 10^6^ cells/well) were seeded in a 96-well plate, and treated with polysaccharides for 24 h. Treatment with lipopolysaccharide (LPS; 1 µg/mL) (Sigma-Aldrich, St. Louis, MO, USA) served as a positive control. After incubation, an equal volume of the Griess reagent was mixed with the supernatants. The absorbance was read at 540 nm using a microplate reader. NO production was calculated using a standard curve.

### 2.9. Gene Expression by RT-PCR

The RAW264.7 cells were seeded in a 24-well plate at a density of 1 × 10^6^ cells/well, and incubated with samples or the LPS stimulant. After 24 h, the cells were extracted using the TRIzol reagent (Invitrogen, Carlsbad, CA, USA). The concentration of RNA was measured with a spectrophotometer before constructing cDNA with an oligo-(dT)20 primer, and Superscript III RT (Invitrogen). The resulting cDNA was amplified by PCR using GoTaq Flexi DNA Polymerase (Promega, Madison, WI, USA). Reverse transcriptase amplification was conducted with initial denaturation, denaturation, annealing, and extension, followed by a final extension step. The PCR product gels were viewed under UV transillumination. The nucleotide sequences of the primers used were as previously reported (Table S1) [32].

### 2.10. Western Blot Analysis

Western blotting was performed according to standard procedure. The RAW264.7 cells were seeded at 1 × 10^6^ cells/well in a 6-well plate, and then treated with LPS or samples for 6 h. The RAW264.7 cells were lysed in RIPA buffer (Tech, and Innovation, Chuncheon, Gangwon, South Korea) containing Inhibitor Cocktail (HaltTM protease, and phosphatase). Cell lysates were separated through 10% SDS-PAGE, and transferred onto PVDF membranes. The membranes were subsequently blocked in 5% non-fat skimmed milk (prepared in Tris-buffer saline containing Tween-20, TBST) at room temperature for 1 h, and incubated with primary antibodies including β-actin (1:1000), anti-phospho-NF-κB (1:1000), anti-phospho-ERK (1:1000), anti-phospho-p38, and anti-phospho-JNK (1:1000) at 4 °C overnight. The membranes were washed with TBST, and incubated with HRP-conjugated anti-rabbit antibody for 1 h at room temperature. Protein was detected using the Pierce^®^ECL Plus Western Blotting Substrate (Thermo Scientific, Waltham, MA, USA) in accordance with the manufacturer’s instructions (Table S2). The bands were obtained using Image Lab software under a ChemiDocTM imaging system.

### 2.11. Investigation of Cell Membrane Binding Receptors

The RAW264.7 cells were adjusted to a concentration of 1 × 10^5^ cells/well in a 96-well plate. The cells were pre-treated with antibodies (anti-TLR4, and anti-CR3, 30 µg/mL) for 2 h, and treated with polysaccharides (200 µg/mL) [36]. The level of NO from the RAW264.7 cells was investigated using the methods described above.

### 2.12. Statistical Analysis

All experiments were repeated at least three times. The data values are expressed as the mean ± SD (n ≥ 3). Statistical analysis was performed using SAS software (SAS Institute, Cary, NC, USA). The differences between groups were analysed using one-way analysis of variance (ANOVA). Significance was defined as a *p*-value of <0.05. Duncan’s multiple range test was used for means comparison.

## 3. Results and Discussion

### 3.1. Yield, and Chemical Composition

As can be seen in Table 2, the total yield of the crude MPs from the mushroom *A. hemibapha* subspecies *javanica* was 4.90%, which was lower than the yield value of polysaccharide from the mushroom *Auricularia auricula-judae* (8.90%) [24]. The discrepancy might be due to the differences in temperature, and time of extraction used [37]. The crude polysaccharides obtained mostly consisted of carbohydrates (87.8%), and were significantly higher than other components, with considerable amounts of protein (6.80%), and sulphate (5.40%). Uronic acids were not found in the MPs. The monosaccharide composition analysis showed that glucose (85.5%) was the main component of the crude polysaccharide, along with small amounts of galactose (6.80%), mannose (3.30%), xylose (1.10%), arabinose (1.80%), and rhamnose (1.40%). Similar results were also found in the crude polysaccharides obtained from *A. strobiliformis* fruiting bodies, glucose being the major sugar, followed by galactose, along with trace amounts of mannose, and xylose [38]. The major sugar content was slightly different from that of the polysaccharide from *A. caesarea*, consisting of glucose, and xylose [39]. It has been reported that mushroom polysaccharides from different species, sources, and growing conditions have different sugar compositions [40].

**Figure 1:**
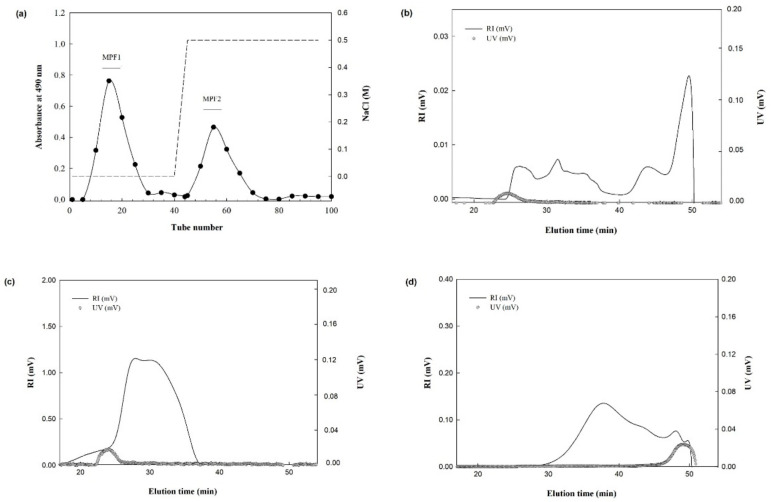
Elution profile of fractionated polysaccharide from *A. hemibapha* subspecies *javanica* through anion-exchange chromatography column (**a**). UV and RI chromatograms of MP (**b**), fraction MPF1 (**c**), and fraction MPF2 by using the size-exclusion chromatography column (**d**).

The MPs were further separated using anion-exchange chromatography to obtain mucilage polysaccharide fractions (MPFs). The MP was eluted with distilled water to obtain a neutral polysaccharide, and subsequently eluted with a stepwise NaCl gradient (0.5–2.0 M). In total, two major fractions were identified as MPF1, and MPF2, with yields of 57.3%, and 42.7%, respectively (Figure 1a). The yield percentage was calculated based on 100% of the MP using the following formula as given below Table 2.

MPF1, and MPF2 mainly consisted of carbohydrates (93.2%, and 83.5%), and MPF1 contained a trace amount of proteins (5.40%), and sulphates (1.40%). The small number of sulphates with the weakest ionic interactions started to elute from the column first by distilled water. However, a trace number of proteins (5.40%) contained in MPF1 might not have been a negative charge of the protein, where they cannot bind to the positively charged matrix on the anion exchangers. In contrast, MPF2 had numbers of protein (7.20%), and sulphates (9.30%) (Table 2). It was eluted with 0.5 M NaCl. The elution that was done by 0.5 M NaCl might be from the tightness of the binding between the negative charges of sulphates, and the positively charged matrix in the anion-exchange column.

MPF1, and MPF2 mainly contained glucose as the major sugar unit. Both fractions contained small amounts of galactose, mannose, xylose, arabinose, and rhamnose. The polysaccharides from other mushroom species, such as *Polyporus rhinocerus*, contain high levels of glucose (87.8%), and mannose (12.2%) [41]. However, the fractionated polysaccharide (VHPI-a) from *Volvariella volvacea* consists of only glucose [27].

### 3.2. Molecular Characteristics of MPs, and MPFs

The chromatograms from the RI, and UV detectors for the MPs, and MPFs are shown in Figure 1b–d. The MPs were eluted from the SEC column at elution times from 25 to 50 min, with broad scattering peaks from the RI chromatogram, indicating their heterogeneous polymer distributions (Figure 1b). As shown in the UV chromatogram, proteins were detected at an elution time of 25 to 28 min. The MW value of the peaks obtained from the multi-angle laser light scattering technique was 479.4 × 10^3^ g/mol (Table 2). The approximate size of the crude mucilage polymer was obtained by the *Rg* calculated from the peaks (Table 2). The *Rg* value of the peaks of crude MP was 148.2 nm.

The chromatogram (RI) of fraction MPF1 showed a major peak at an elution time of 25 to 35 min, and no UV chromatogram was shown for MPF1 (Figure 1c), which is in good agreement with the protein content presented in Table 2. The MW was 219.2 × 10^3^ g/mol, and the *Rg* value was 132.3 nm. For fraction MPF2, the RI, and UV chromatograms were shown to be in the same direction (Figure 1d), RI revealing elution times of 30 to 50 min, and UV showing an elution time of 47 to 50 min. The MW, and *Rg* values were 104.0 × 10^3^ g/mol, and 173.1 nm, respectively (Table 2).

Thus, we obtained two low molecular weight fractions (MPF1; 219.2 × 10^3^ g/mol, and MPF2; 104.0 × 10^3^ g/mol) from MP (479.4 × 10^3^ g/mol). Similarly, the low molecular weight fractions were obtained after fractionation by using anion-exchange chromatography [42,43]. As reported, the polysaccharides extracted from *Myriophyllum spicatum* L., and fractionated using the DEAE Sepharose fast flow column exhibited the crude MW, and two fractions as 529.0, 497.8, and 217.4 × 10^3^ g/mol [44]. Moreover, the polysaccharide from *Gracilaria rubra* (GRPS), and its three purified fractions GRPS-1-1, GRPS-2-1, and GRPS-3-2 were obtained through water extraction, and ion chromatographic purification. The three fractions showed the MW, 1310, 691, and 923 × 10^3^ g/mol [45]. Moreover, it is also reported in the literature that polysaccharides with MW > 200 × 10^3^ g/mol are good immunogens, and demonstrate immunostimulatory properties. Lentinan, and schizophyllan have a MW of 300–800 and 450 × 10^3^ g/mol, respectively [46].

### 3.3. Immunomodulatory Activity of MPs, and MPFs

**Figure 2:**
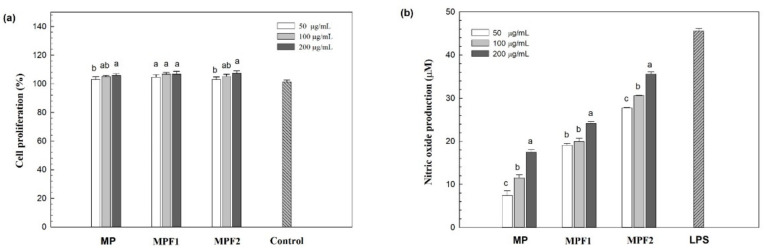
Effect of MP, MPF1, and MPF2 on RAW264.7 cell activity. RAW264.7 cell proliferation with different polysaccharide treatment concentrations. Medium alone was used as a control (**a**). Nitric oxide (NO) production, and the medium alone was used as a control, while LPS was used as the positive control (**b**). Values are presented as mean ± standard deviation (n = 3). Different letters a,b,c indicate significant differences (*p* < 0.05) among the groups in concentration.

The effects of MPs, and MPFs on immunomodulation were tested in RAW264.7 murine macrophage cells. Macrophage cells are well known to be closely related to the response of both the innate, and adaptive immune systems by releasing NO or several cytokines such as IL-6, IL-10, IL-1β, and TNF-α [30]. Generally, mushroom β-glucans, and/or α-glucans with the structure linear, and/or branched effect on the immune system function mainly through macrophages stimulation. These β/or α-glucans could bind to specific receptors such as Toll-like receptors (TLRs) of the macrophage to trigger the immune response by releasing nitric oxide (NO), and producing various cytokines such as TNF-α, IL-6, and IL-1β [27]. The cell proliferation stimulated by the polysaccharides (MP, MPF1, and MPF2) was determined in order to evaluate the cytotoxic effect at a concentration of 50–200 µg/mL (Figure 2a). Incubation of the macrophage cells with the crude extract, and fractions (MP, MPF1, and MPF2) considerably improved proliferation of the RAW264.7 cells compared to the control, which suggests that the crude extract, and fractions are non-toxic to the cells at the tested concentrations.

The biological mediators, such as NO, released from the RAW264.7 cells by MP, MPF1, and MPF2 at concentrations of 50–200 µg/mL are presented in Figure 2b. Approximately 7.37–17.4 µM NO was released by the MP at a concentration of 50–200 µg/mL. Similarly, MPF1 stimulated the secretion of NO to around 19.0–24.1 µM. Fraction MPF2 exhibited the highest NO production: >27 µM at all concentrations. The NO production induced by treatment with MPF2 was comparable to that for the positive control (LPS; 1 µg/mL), suggesting its strong stimulation of macrophage cells. The NO production of the RAW264.7 cells was in agreement with that reported for treatment with *V. volvacea* in the range of 7.76–32.5 µg/mL [27], and 2–13 µg/mL for *Armillariella tabescens* [47]. It has been reported that the key factors affecting immune activity, such as MW, polymer structure, and chemical compositions, which include protein or sulphate affect immunostimulatory properties. As reported by Surayot et al. (2014), the low MW of polysaccharides from lactic acid bacteria provides better immunostimulation of immune cells [48]. In addition, the sulphate polysaccharides from *Hypsizigus marmoreus*, and *Cladophora glomerata* Kützing exhibit strong production of NO, and various cytokines [33,49]. In this study, MPF2 had a lower MW than MP, and MPF1, which seems to be related to NO stimulation. The correlation between the structure, and biological activity of polysaccharides is too complicated to elucidate. Future research studies on the structure, and activity relationship are currently being conducted.

### 3.4. Effect of MP, and MPF Stimulation on Cytokine mRNA Expression

**Figure 3:**
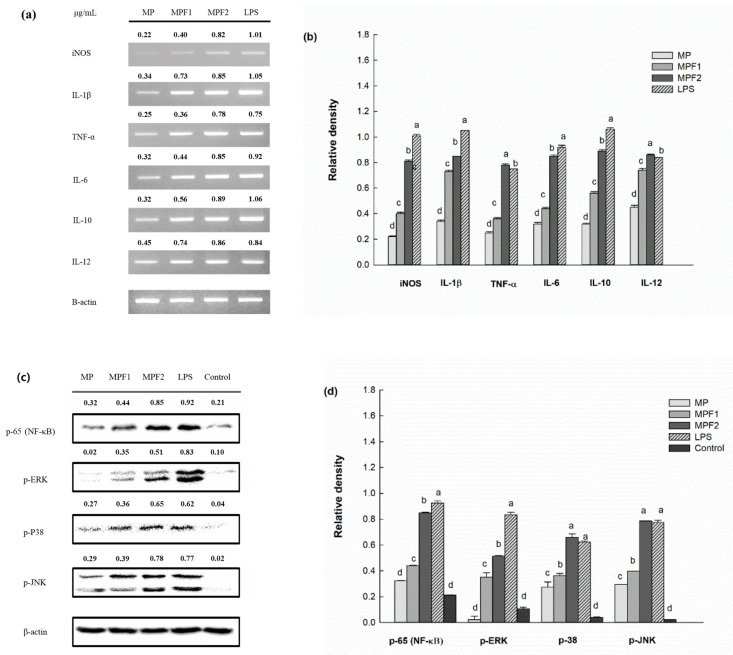
Effects of MP, MPF1, and MPF2 from *A. hemibapha* subspecies *javanica* on cytokine mRNA expression in RAW264.7 cells (**a**), and the relative band intensity (**b**). Phosphorylation of p65, ERK, P38, and JNK by MP, MPF1, and MPF2 (**c**), and the relative band intensity (**d**). Different letters a,b,c,d indicate a significant difference (*p* < 0.05) among the MP, MPF1, and MPF2.

The mRNA expression of inducible nitric oxide synthase (iNOS) in the RAW264.7 cells induced by MP, MPF1, and MPF2 was determined by RT-PCR. As shown in Figure 3a,b, after the addition of the sample, cells treated with MPF2 showed a strong band, which indicated the marked induction of iNOS gene expression. Therefore, the increased levels of NO released from the RAW264.7 cells might be associated with enhanced mRNA, and protein expression of iNOS that appeared to be upregulated in the RAW264.7 cells through activation by the fraction MPF2.

The mRNA expression of pro-inflammatory cytokines, including IL-1β, TNF-α, and IL-6, in the RAW264.7 cells is shown in Figure 3a,b. The mRNA expression of IL-1β, TNF-α, and IL-6 induced by MPF2 could stimulate the RAW264.7 cells to produce a considerable amount of cytokines. In this research, IL-10, and IL-12 were also noticed after treatment with MP, and MPFs to conquer excess production of pro-inflammatory cytokines. Therefore, the polysaccharide of *A. hemibapha* subspecies *javanica* might be capable of modulating the host immune system by releasing various cytokines. Similar results were seen in polysaccharides from *C. cibarius*, which stimulated RAW264.7 cells by secretion of TNF-α, IL-1β, IL-2, IL-6, IL-10, and IL-12 [16].

### 3.5. Immunomodulatory Mechanism by Western Blot Analysis

Further study was performed to elucidate the mechanism by which the polysaccharides stimulate the production of inflammatory mediators such as cytokines, and NO in macrophage cells; thus, nuclear factor-kappa B (NF-κB), and MAPK activation was investigated. NF-κB is a major transcription factor, and regulates many target genes, playing an important role in immunomodulatory activity. In the activated condition, phosphorylation of the inhibitor (I-κB) results in NF-κB translocation to the nucleus. After being translocated to the nucleus, NF-κB starts the transcription of the target genes, which encode various cytokines, and inducible enzymes. As shown in Figure 3c,d, MP, MPF1, and MPF2 were tested at 200 µg/mL. The phosphorylation of NF-κB (p65) was significantly higher in treatments with MPF2 than MP, and MPF1, indicating that the MPF2 induces the translocation of NF-κB (p65) from the cytosol to the nucleus, which results in the activation of RAW264.7 cells. In addition, the MAPK pathways play a role in the regulation of immune responses. MAPK members such as ERK, JNK, and p-38 are shown in Figure 3. Treatment with MPF2 strongly induced the phosphorylation of ERK, p38, and JNK compared with the control. Overall, the polysaccharides of *A. hemibapha* subspecies *javanica* stimulated the RAW264.7 cells via activation of the NF-κB, and MAPK pathways. This results in agreement with the report by Cui et al. (2020) regarding the fact that the polysaccharide (glucan) from the fruiting body of *V. volvacea* also activates macrophage cells via the MAPK pathways [27].

### 3.6. MPF2 on Binding Receptors

**Figure 4:**
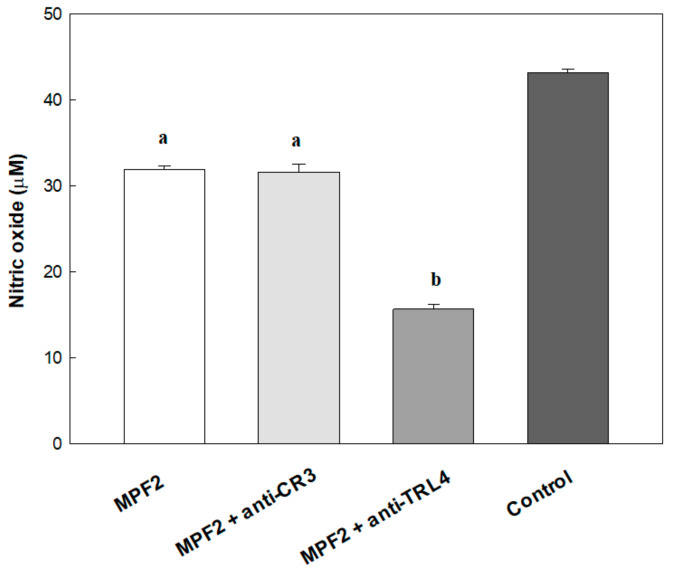
Nitric oxide (NO) production in the antibody neutralisation assay. RAW264.7 cells were pre-incubated with antibodies of the receptors for 2 h before stimulation with MPF2. LPS was used as the positive control. Data are presented as mean ± standard deviation. Different letters a,b indicate a significant difference (*p* < 0.01) between polysaccharides (MPF2), MPF2 + anti-CR3 Ab, and MPF2 + anti-TLR4 Ab.

It has been found that polysaccharide from mushroom activates macrophages through the activation of pathogen-associated molecular patterns (PAMPs) by pattern recognition receptors (PRRs) on the surface of cells. Some evidence shows that the polysaccharides might bind to cell surface receptors such as complement receptor 3 (CR3), and toll-like receptor 4 (TLR4) [50]. In the present study, the role of TLR4, and CR3 was investigated, as shown in Figure 4. The RAW264.7 cells were pre-treated with 30 µg/mL of anti-TLR4, and anti-CR3 for 2 h; after blocking the binding receptors, the level of NO production was significantly decreased by anti-TLR4 but not anti-CR3, therefore suggesting that the polysaccharide from *A. hemibapha* subspecies *javanica* (MPF2) stimulated the RAW264.7 cells via activation of the NF-κB, and MAPK pathways through the binding of TLR4 receptors (Figure S1). These results agree with the study conducted by Hsu et al. (2004) who showed that the extract of *Ganoderma lucidum* polysaccharides shows immunomodulatory activity by stimulating the expression of cytokines via the recognition of TLR4 [51].

### 3.7. Structural Analysis of MPF2

**Figure 5:**
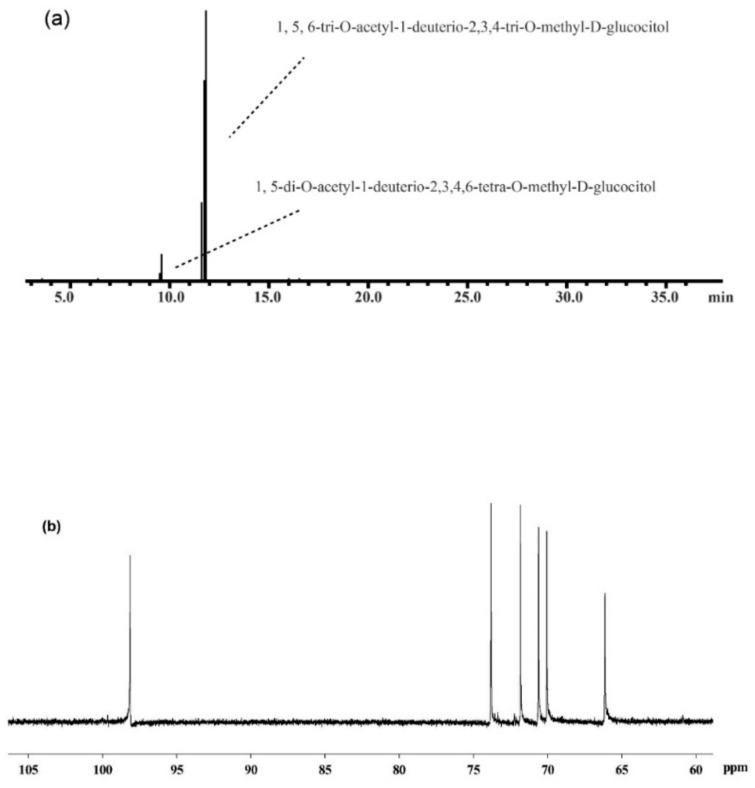

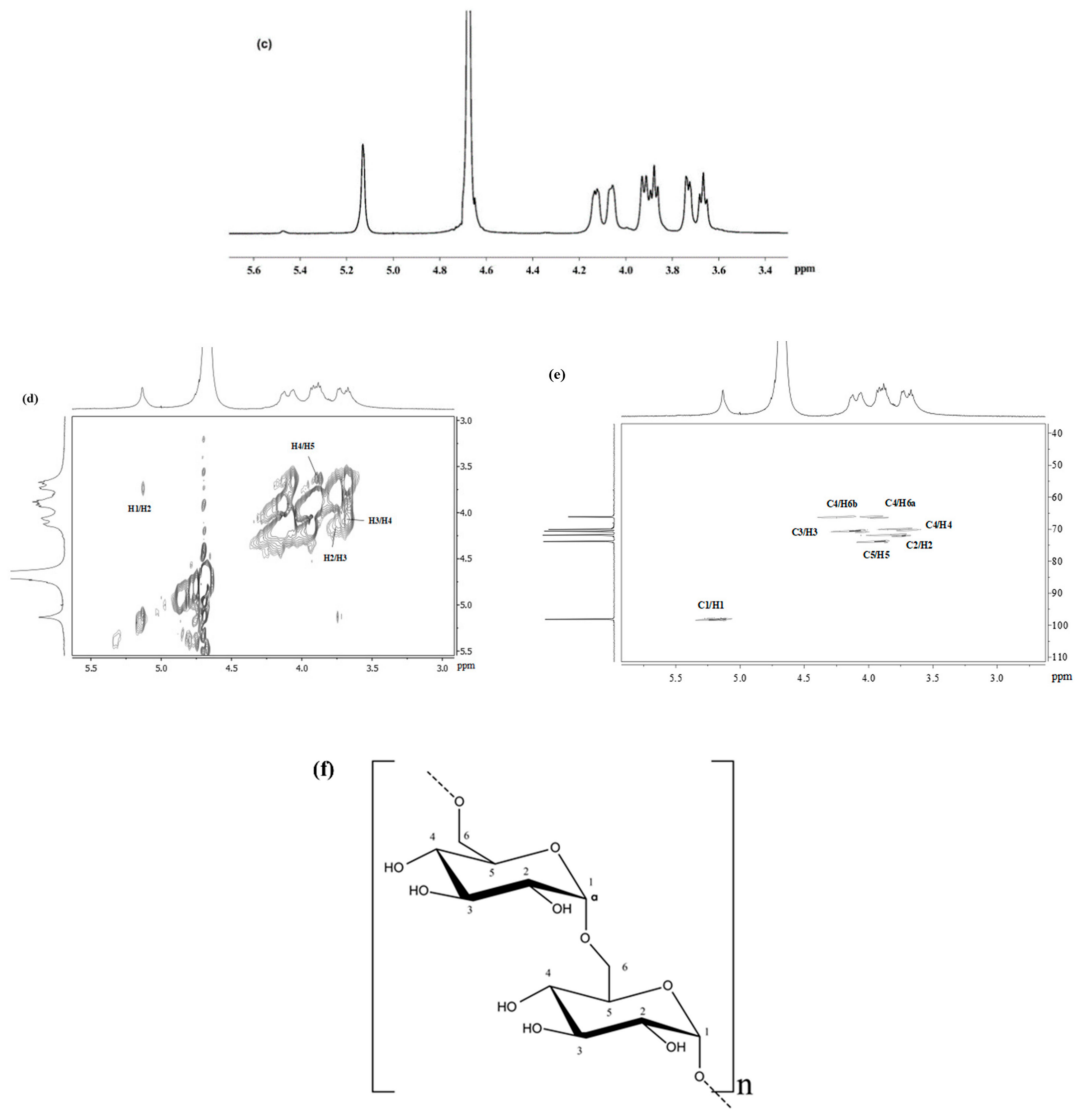
GC–MS spectra (**a**), ^13^C-NMR spectra (**b**), ^1^H-NMR spectra (**c**), COSY-NMR spectra (**d**), HMQC-NMR spectra (**e**), and the main backbone of MPF2 (**f**) from *A. hemibapha* subspecies *javanica*.

The most immunoenhancing polysaccharide (MPF2) was subjected to structural analysis by GC–MS, and 1D-NMR spectral analysis to elucidate the glycosidic linkages. The glycosidic linkages of MPF2 were elucidated by methylation analysis as shown in Table 3. The most abundant components of the partially methylated, and acetylated derivatives were 2,3,4-trimethyl-Glcp, which implies that the backbone of the MPF2 fraction was mainly glucose connected by (1→6) linkages (Figure 5a). The absolute configuration of the monosaccharide indicated that glucose is present in the D configuration.

Furthermore, glycosidic linkages of MPF2 were confirmed by 1D, and 2D-NMR analysis. As shown in Figure 5b,c, and Table 4, only one anomeric signal at 97.2, and 5.19 ppm appeared in the ^13^C NMR, and ^1^H spectra. The ^1^H anomeric chemical shift at 5.19 ppm had a small value of the ^3^J_1,2_ coupling constant (~3.04), indicating that it is an α-anomeric configuration. The other ^1^H signals using the COSY spectrum (Figure 5d) were at 3.75, 3.98, 3.72, 4.14, and 3.95/4.19, which were ascribed as H-2, H-3, H-4, H-5, and H-6a/b, respectively. Conversely, the other carbon signals (Figure 5e) were assigned by the HMQC spectrum, marked at 72.3, 70.7, 70.0, 74.1, and 66.3 for C-2, C-3, C-4, C-5, and C-6, respectively. The C-6 peak showed a downfield shift of 66.3 ppm due to the effect of glycosylation, which is comparable to the resonance of a glucose standard [52]. Thus, MPF2 is an α-(1→6)-linked-glucopyranosyl moiety. After the comprehensive methylation analysis, and NMR analysis, it can thus be concluded that the backbone of MPF2 is α-(1→6)-linked-glucopyranosyl (Figure 5f). The glycosidic linkages were similarly studied in polysaccharides from the mushroom *A. tabescens*, exhibiting a main backbone of α-(1→6)-linked-glucan [47]. In contrast, the structure of polysaccharides from mushrooms from other species are of different types [28,53].

## 4. Conclusions

The MP from *A. hemibapha* subspecies *javanica* (Corner, and Bas) was purified using a DEAE–Sepharose fast flow column. Fraction MPF2 had a homogenous molecular distribution with an average MW of 104.0 × 10^3^ g/mol. The chemical constituents of MPF2 were primarily glucose, and a minor amount of rhamnose, arabinose, mannose, and galactose. The highest levels of NO, and pro-inflammatory cytokines were secreted from RAW264.7 cells treated with MPF2. The interaction of RAW264.7 cells with MPF2 occurred through the cell surface receptor TLR4, initiating the cascade activation of the NF-κB, and MAPK pathways. The structural backbone of MPF2 mainly consists of α-(1→6)-linked-glucopyranosyl residues. The result of this study demonstrates the important role of MPF2 in the development of new immunostimulants for application in novel functional foods, and nutraceuticals. Further research on the binding mechanism between polysaccharides, and cell surface receptors would lead to a better understanding of the relationship between its molecular structures, functional groups attached to polysaccharide, and immunomodulation activities through investigating in vitro (macrophage, and NK cell lines), and in vivo (mice, and rats). This research is required prior to applying MPF2 in food products and determining its stability for industrial food or nutraceutical applications.

## Figures and Tables

**Table 1:** Extraction methods, and bioactivities of polysaccharide from mushrooms.

Polysaccharide	Bioactivity	Extraction Method	References
*Cantharellus cibarius*	Immunomodulatory activity	Boiling water extraction	[16]
*Flammulina velutipes*	Immunomodulatory, and antioxidant activity	Hot water extraction (80 °C), and ultrasound-assisted extraction	[17]
*Cordyceps sinensis*	Anticancer, and antioxidant activity	Hot water extraction (95–100 °C)	[18,21]
*Lepista nuda*	Antioxidant, antitumor, and antiviral activities	Water extraction (75–95 °C)	[19]
*Lentinus edodes*	Reno-protective activity	Water extraction (85 °C)	[20]
*Collybia radicata*	Immunomodulatory activity	Water extraction (84 °C)	[22]
*Ganoderma lucidum*	Anti-inflammatory	Boiling water extraction	[23]
*Auricularia* *auricula-judae (Bull.)*	Skin wound-healing	Water extraction (97 °C)	[24]

**Table 2:** Extraction methods, characterisation, and bioactivities of polysaccharide from edible mushroom of *A. hemibapha* subspecies *javanica*.

	Polysaccharides		
Component	MP	MPF1	MPF2
Yield (%)	4.90 ^*x*^	57.3 ^*y*^	42.7 ^*y*^
Total carbohydrate (% db)	87.8 ± 2.64 ^b^	93.2 ± 0.12 ^a^	83.5 ± 0.50 ^c^
Protein (% db)	6.80 ± 0.17 ^b^	5.40 ± 0.20 ^c^	7.20 ± 0.09 ^a^
Sulphate (% db)	5.40 ± 0.20 ^b^	1.40 ± 0.03 ^c^	9.30 ± 0.10 ^a^
Uronic acid (%)	-	-	-
Monosaccharide content (%)			
Rhamnose	1.40 ± 0.05 ^b^	2.70 ± 0.03 ^a^	0.80 ± 0.10 ^c^
Arabinose	1.80 ± 0.08 ^b^	4.70 ± 0.08 ^a^	0.30 ± 0.02 ^c^
Xylose	1.10 ± 0.10 ^b^	2.50 ± 0.20 ^a^	-
Mannose	3.30 ± 0.30 ^a^	1.40 ± 0.10 ^b^	0.30 ± 0.00 ^c^
Glucose	85.5 ± 0.50 ^b^	79.6 ± 1.35 ^c^	98.4 ± 0.46 ^a^
Galactose	6.80 ± 2.64 ^b^	9.10 ± 0.00 ^a^	0.20 ± 0.02 ^c^
Molecular properties			
Samples	MW × 10^3^ (g/mol)	*Rg* (nm)	
MP	479.4 ± 14.7 ^a^	148.2 ± 10.0 ^a^	
MPF1	219.2 ± 14.8 ^b^	132.3 ± 6.4 ^b^	
MPF2	104.0 ± 13.1 ^c^	173.1 ± 5.9 ^c^	

^*x*^ Yield, (weight of crude/weight of sample powder) × 100. ^*y*^ Yield, (weight of fractions/weight of crude injected into column) × 100. % db, % dry basis. Abbreviations: MW, mean average molecular weight, and *Rg*, radius of gyration. Different letters indicate significant differences (*p* < 0.05) among the groups in each row for total carbohydrate and monosaccharide content, and each column for molecular properties.

**Table 3:** Glycosidic linkage analysis of the most immunostimulating polysaccharide (MPF2) from *A. hemibapha* subspecies *javanica*.

Methylation Sugar	Retention Time (min)	Mass Fraction (m/z)	Glycosidic Linkage	Peak Area (%)
2,3,4,6-Me_4_-Glc	9.52	43, 59, 71, 87, 102, 118, 129, 145, 162, 174, 205	Glc-(1→	1.7
2,3,4-Me_3_-Glc	11.8	43, 59, 71, 87, 102, 118, 129, 162, 174, 189, 233	→6)-Glc-(1→	98.3

**Table 4:** ^1^H, and ^13^C NMR spectral data of the most immunostimulating polysaccharide (MPF2) from *A. hemibapha* subspecies *javanica*.

Residue	H-1/C-1	H-2/C-2	H-3/C-3	H-4/C-4	H-5/C-5	H-6a, H-6b/C-6
α-D-(1→6) Glcp	5.19/97.2	3.75/72.3	3.98/70.7	3.72/70.0	4.14/74.1	3.95, 4.19/66.3

## Data Availability

Any additional data can be available upon request to the corresponding author.

## Supplementary Materials

The following are available online at https://www.mdpi.com/article/10.3390/jof7090683/s1, Table S1: The sequences of primer used for RT-PCR, Table S2: Enhanced chemiluminescence (ECL) kit, and its instructions, Figure S1: a schematic illustration for the interaction of MPF2 to TLR4.
