# Supplementary material for: Extraction, Structural Characterisation, and Immunomodulatory Properties of Edible Amanita hemibapha subspecies javanica (Corner and Bas) Mucilage Polysaccharide as a Potential of Functional Food
Source: J Fungi (Basel). 2021 Aug 24;7(9):683. doi: 10.3390/jof7090683 (PMC8468940; doi:10.3390/jof7090683)
Supplement: Supplementary file 1 [file jof-07-00683-s001.zip › jof-1349367-supplementary.pdf]

Table S1. The sequences of primer used for RT-PCR.

Gene		Primer sequences (5'→3')
iNOS	Forward	CTGCAGCACTTGGATCAGGAACCTG
	Reverse	GGGAGTAGC CTGTGTGCACCTGGAA
IL-1β	Forward	ATGGCAACTATTCCAGAACTCAACT
	Reverse	CAGGACAGGTATAGATTCTTTCCTTT
TNF-α	Forward	AGGTTCTGTCCCTTTCACTCACTG
	Reverse	AGAAGACCTGGGAGTCAAGGTA
IL-6	Forward	TTCCTCTCTGCAAGAGACT
	Reverse	TGTATCTCTCTGAAGGACT
IL-10	Forward	TACCTGGTAGAAGTGATGCC
	Reverse	CATCATGTATGCTTCTATGC
IL-12	Forward	CCACAAAGGAGGCGAGACTC
	Reverse	CTCTACGAGGAACGCACCTT
β-actin	Forward	ATGTGCAAAAAGCTGGCTTTG
	Reverse	ATTTGTGGTGGATGATGGAGG

Table S2. Enhanced chemiluminescence (ECL) kit and its instructions.

**ECL kit instructions**
1. Prepare working solution of western blot detection by mix the solution A and B in the ratio of 40:1 (390 μl of Solution A + 10 μl of Solution B)
2. Incubate the membrane with detection buffer for 5 min
3. Drain excess reagent and cover the blot with clear film
4. Detect the signal by ChemiDocTM imaging system

- The signals were measured using Pierce®ECL Plus Western Blotting Substrate (Thermo Scientific, Waltham, MA, USA).

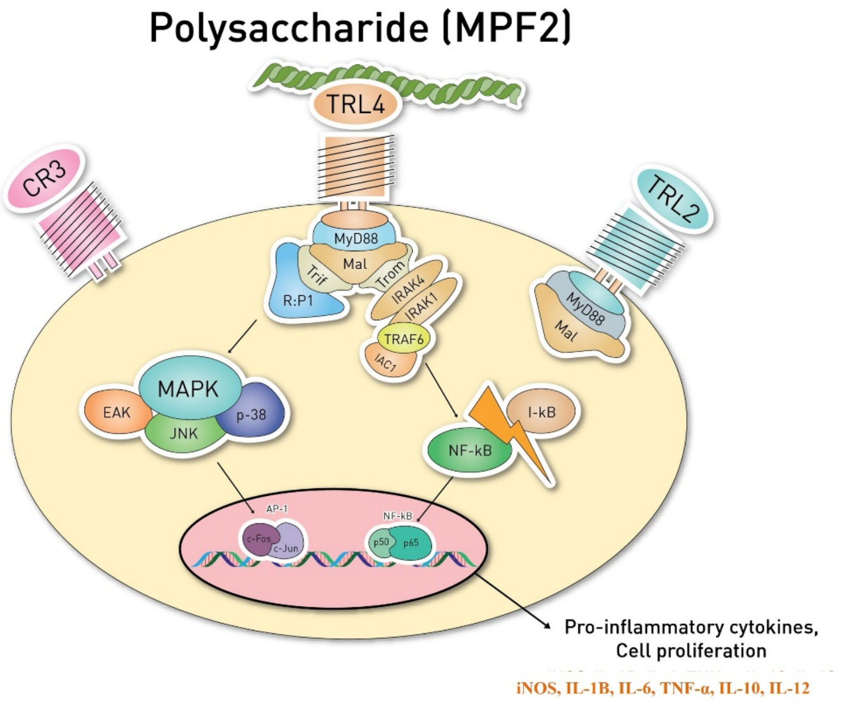

Figure S1. A schematic illustration for the interaction of MPF2 to TLR4.
